# Midwife-led continuity education on fear and birth outcomes in primiparas

**DOI:** 10.1590/1806-9282.20252084

**Published:** 2026-06-29

**Authors:** Özlem Koç, Şule Gökyıldız Sürücü

**Affiliations:** 1 Tarsus University, Faculty of Health Sciences, Department of Midwifery – Mersin, Türkiye.; 2 Çukurova University, Faculty of Health Sciences, Department of Midwifery – Adana, Türkiye.

**Keywords:** Trauma and stressor related disorders, Psychological, Delivery, Continuity of care, Educational model, Midwifery

## Abstract

**OBJECTIVE:**

The aim of this study was to examine the effects of midwife-led continuity education on fear of childbirth, mode of delivery, and postpartum trauma among primiparous women.

**METHODS:**

A single-blind randomized controlled trial was conducted at Adana City Hospital, Turkey, between December 2023 and November 2024. Ninety-two primiparous pregnant women were enrolled and randomized to an intervention group (n=46) or a control group (n=46). Inclusion criteria were age 18–35 years, singleton low-risk pregnancy, Turkish literacy, and no medical or obstetric contraindications to vaginal birth. Women with high-risk pregnancies, psychiatric disorders, systemic diseases, or prior structured childbirth education were excluded. The intervention group received a four-module education program based on the Midwife-Led Continuity of Care Model, while the control group received routine antenatal care.

**RESULTS:**

Baseline childbirth fear scores were comparable between groups. Following the intervention, mean childbirth fear scores were lower in the intervention group than in the control group. One month postpartum, mean City Birth Trauma Scale scores were 5.43±5.69 in the intervention group and 22.93±9.92 in the control group. Vaginal birth occurred in 69.6% of women in the intervention group compared with 24.4% in the control group. Among women who had vaginal births, postpartum fear scores remained lower in the intervention group.

**CONCLUSION:**

Midwife-led continuity education is associated with reduced fear of childbirth, lower postpartum trauma symptoms, and higher vaginal birth rates among primiparous women. These findings support the integration of continuity-based midwifery education into routine maternity care.

## INTRODUCTION

Every woman has the right to respectful, individualized, and continuous care throughout pregnancy and childbirth, ensuring maternal and neonatal well-being^1^. The World Health Organization (WHO) highlights that positive birth outcomes depend not only on clinical safety but also on woman-centered care, participation, and emotional support during labor^2^.

Turkey has one of the highest cesarean section rates among Organization for Economic Co-operation and Development (OECD) countries, reaching 54.4% in 2019^3^. Fear of childbirth, negative perceptions of vaginal birth, and insufficient emotional support contribute to this preference^4,5^. Although national guidelines recommend at least four antenatal visits, follow-ups often remain limited and do not provide holistic care^6^.

The Midwife-Led Continuity of Care Model (MLCCM) emphasizes respect, autonomy, and relationship-based continuity between a woman and her midwife^7,8^. Evidence shows that MLCCM supports physiologic birth, reduces interventions, enhances satisfaction, and improves maternal mental health^9-12^.

In Turkey, implementation remains limited. This study evaluates the effects of MLCCM-based education on primiparous women's childbirth fear, birth preferences, and postpartum trauma perceptions, aiming to provide evidence for integrating midwife-led continuity into national maternity services.

## METHODS

### Study design

This single-blind, randomized controlled longitudinal trial was conducted at Adana City Hospital, Turkey, between December 2023 and November 2024. The study evaluated the effects of midwife-led education within the MLCCM on primiparous women's childbirth fear, mode of delivery preferences, and postpartum trauma perceptions. Consolidated Standards of Reporting Trials (CONSORT) 2010 guidelines were followed^13^.

### Participants and sample size

A total of 92 primiparous pregnant women (46 intervention and 46 control) receiving routine prenatal care were included. Inclusion criteria were age 18–35 years, at least primary education, Turkish literacy, singleton healthy pregnancy, and no obstetric or medical contraindications to vaginal birth. Exclusion criteria included high-risk pregnancy, systemic or psychiatric disorders, and prior childbirth education.

Sample size was calculated using previous studies^14^ with α=0.05 and 99% power, resulting in 82 participants; allowing for a 10% attrition rate, 92 participants were recruited. Randomization was performed by an independent statistician using a computer-generated block randomization method.

### Randomization

Participants were randomly allocated to the intervention or control group using a computer-generated block randomization method. Randomization was performed by an independent statistician who was not involved in participant recruitment, intervention delivery, or outcome assessment.

### Blinding

This study was designed as a single-blind trial. Due to the nature of the educational intervention, participants could not be blinded to group allocation. However, outcome assessments and statistical analyses were performed by personnel who were blinded to the intervention and control groups.

### Intervention

**Figure 1:**
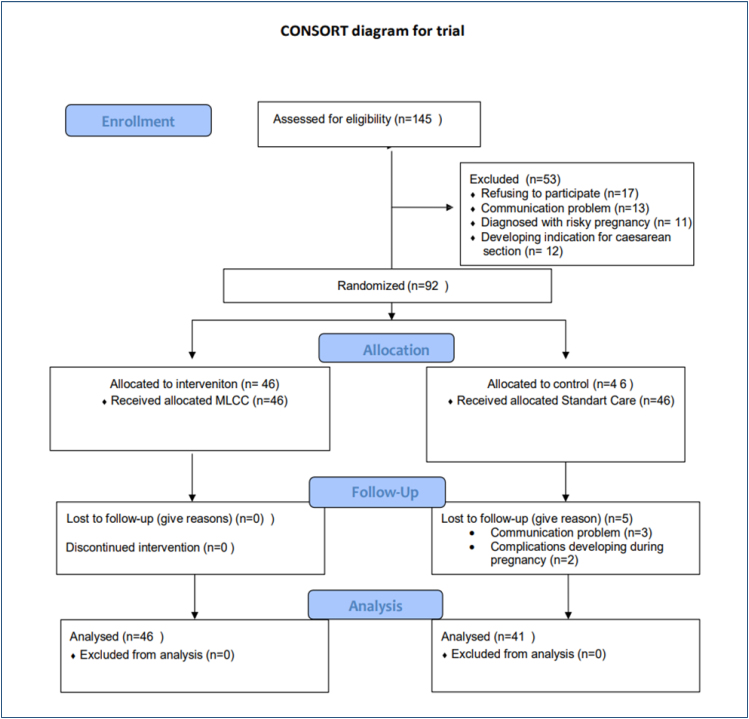
Consolidated Standards of Reporting Trials flow diagram of the randomized controlled trial (adapted from the Consolidated Standards of Reporting Trials 2010 Statement)^13^.

The intervention group received a four-module education program based on MLCCM and WHO antenatal–postnatal care recommendations, validated by an expert panel (Table 1). Modules covered pregnancy adaptation (weeks 20–28), fear management and preparation for motherhood (29–36), labor process and benefits of vaginal birth (37–40), and postpartum care with a home visit (first month) (Figure 1). Each session (∼90 min) was delivered by the same midwife to maintain relational continuity. The control group received routine care only.

**Table 1:** Training content of the four-module midwife-led continuity of care model.

Module	Content	Process	Method
Module 1 week 20–28	• Welcome and introduction• Physiological changes during pregnancy• Complaints during pregnancy and solutions• Signs of danger in pregnancy and what to do• Antenatal tests and their importance• Nutrition, exercise, and sexuality in pregnancy• Common misconceptions about pregnancy• Adaptation to pregnancy• Medical procedures in hospital controls	Session 1 — 30 min narration+10 min break Session 2 — 30 min narration+10 min break Session 3 — 30 min narration+10 min Q&A	Face-to-face education
Module 2 week 29–36	• How fear affects childbirth• Management of birth waves• True and false labor pains• Importance of breathing exercises• Infant care demonstration• Breastfeeding and lactation• Sleep and rest during pregnancy• Adaptation to motherhood• Preparing mother and baby bag		
Module 3 week 37–40	• Signs of labor onset• Vaginal birth process• Breathing practice• Family planning counseling• Importance of breastfeeding• Preparing for puerperium• Ideal birth environment		Online education
Module 4 postpartum 1st month	• Breastfeeding support• Infant care• Immunization• Maternal self-care• Mother–infant bonding• Postpartum blues• Neonatal jaundice information• Family planning counseling	60 min hands-on individualized care based on needs	Face-to-face education

### Data collection tools

Data were collected using three validated instruments. The Wijma Delivery Expectancy Questionnaire (W-DEQ Version A), developed by Wijma et al.^15^ and adapted into Turkish by Korukcu et al.^16^ (Cronbach's α=0.89), measured antenatal childbirth fear; α=0.93 in this study. The W-DEQ (Version B), developed by Wijma et al.^17^ and validated in Turkish by Ucar and Beji^18^ (α=0.88), assessed postpartum experiences; α=0.95 in this study. The City Birth Trauma Scale, developed by Ayers et al.^19^ and validated in Turkish by Bayrı Bingöl et al.^20^ (α=0.91), assessed post-traumatic stress disorder (PTSD) symptoms; α=0.90 in this study. Pre-tests were completed antenatally (W-DEQ A) and post-tests 1 month postpartum (W-DEQ B and City Birth Trauma Scale [City BiTS]).

### Data collection and analysis

Data were analyzed using International Business Machines Statistical Package for the Social Sciences (SPSS) Statistics version 27.0. Descriptive statistics (mean, standard deviation, frequency, and percentage) were used to summarize participants’ characteristics. The normality of continuous variables was assessed using normality tests and distribution characteristics, and the data were found to be normally distributed. Therefore, parametric tests were applied.

Comparisons of continuous variables between the intervention and control groups were performed using independent samples t-tests, while categorical variables, including mode of delivery, were analyzed using the chi-square test. A p-value of <0.05 was considered statistically significant. An intention-to-treat analysis was not performed; analyses were conducted using a per-protocol approach, including participants with complete outcome data.

### Ethical considerations

Ethical approval was obtained from the university ethics committee (No. 128/64-2/12/2022) and from the institutional authorities of the study site. Written informed consent was obtained from all participants prior to enrollment. The study was conducted in accordance with the principles of the Declaration of Helsinki and was prospectively registered at ClinicalTrials.gov (NCT06020339).

## RESULTS

A total of 92 primiparous women were initially enrolled and randomized (46 intervention and 46 control). Postpartum outcome data were available for all women in the intervention group and for 41 women in the control group. Groups were similar in sociodemographic characteristics (p>0.05), confirming successful randomization.

Baseline childbirth fear scores did not differ significantly (p=0.89). After the MLCCM-based education, fear scores significantly decreased in the intervention group compared with controls (p<0.05). One month postpartum, PTSD symptoms measured by the City BiTS were significantly lower in the intervention group (5.43±5.69) than in the control group (22.93±9.92) (p<0.001).

Mode of delivery differed significantly: 69.6% of women in the intervention group had vaginal births versus 24.4% in the control group (p<0.001). Among vaginal births, W-DEQ B scores were lower in the intervention group (71.53±25.42 vs. 91.50±22.36; p=0.03), indicating sustained fear reduction.

Overall, MLCCM-based education effectively reduced childbirth fear, improved birth outcomes, and minimized postpartum trauma symptoms (Table 2).

**Table 2:** Comparison of childbirth fear, postpartum trauma, and delivery mode outcomes between intervention and control groups.

Outcome	Time of measurement	Intervention n (%)/mean±SD	Control n (%)/mean±SD	p-value
**Fear of childbirth (W-DEQ A)**	Before education	54.46±28.61 (n=46)	53.71 ± 20.37 (n=41)	0.89
**Fear of childbirth (W-DEQ B)** *	1 month postpartum	71.53±25.42 (n=32)	91.50 ± 22.36 (n=10)	**0.03**
**Postpartum trauma (City BiTS)**	1 month postpartum	5.43±5.69 (n=46)	22.93 ± 9.92 (n=41)	**<0.001**
**Mode of delivery** *	Birth	32 (69.6%) vaginal 14 (30.4%) cesarean	10 (24.4%) vaginal 31 (75.6%) cesarean	**<0.001**

Note:

* W-DEQ B and delivery mode comparisons include only women who had vaginal births.

City BiTS was administered 1 month postpartum to all participants. W-DEQ A: Wijma Delivery Expectancy Questionnaire Version A; W-DEQ B: Wijma Delivery Expectancy Questionnaire Version B; SD: standard deviation; City BiTS: City Birth Trauma Scale. Bold values indicate statistically significant differences (p<0.05).

## DISCUSSION

This randomized controlled study demonstrated that education provided within the MLCCM significantly reduced fear of childbirth, decreased postpartum post-traumatic stress disorder symptoms, and increased the rate of vaginal births among primiparous women. These findings reinforce the importance of woman-centered, continuous midwifery care in promoting positive childbirth experiences.

The results are consistent with previous research showing that continuous midwife-led care enhances women's confidence, reduces anxiety, and supports physiological birth^7,11^. Within the MLCCM framework, emotional and relational continuity between the woman and her midwife fosters trust and empowerment—key determinants of reduced fear and improved satisfaction. This aligns with Bandura's self-efficacy theory, which posits that perceived competence and consistent support strengthen coping mechanisms during challenging life events such as labor.

The significant reduction in childbirth fear after the educational intervention parallels earlier findings by Gökçe İsbir et al.^14^ and Serçekuş and Baskale^21^, who reported that structured antenatal education increased self-efficacy and reduced anxiety. Similarly, studies from different settings^22-24^ confirmed that antenatal programs focusing on knowledge, relaxation, and emotional preparation encourage women to choose vaginal delivery.

An important contribution of the present study is its preventive focus on postpartum trauma. While most previous studies addressed post-traumatic stress after birth^25,26^, this trial integrated preventive midwifery care from the antenatal to postpartum period. The significantly lower postpartum PTSD scores in the intervention group demonstrate that continuity of care not only treats but also prevents psychological trauma associated with childbirth.

These findings are particularly relevant in Turkey, where cesarean rates remain among the highest in the OECD^3^. The results suggest that midwife-led continuity of care may reduce unnecessary cesarean births by alleviating fear-driven preferences. Strengthening midwives’ roles and incorporating MLCCM principles into national maternal care policies could promote safer and more satisfying birth experiences.

Methodologically, the study's strengths include its randomized controlled design, longitudinal follow-up, and the use of validated instruments with high reliability (Cronbach's α>0.90). The single-midwife delivery of the educational program ensured relational continuity and minimized variability in intervention quality. The study also followed CONSORT 2010 guidelines and was registered prospectively, enhancing transparency and rigor. In addition, the use of blinded outcome assessment and statistical analysis represents a methodological strength of this study by minimizing potential assessment bias.

However, certain limitations should be acknowledged. The research was conducted in a single tertiary hospital, which may limit generalizability. The involvement of the same midwife as both educator and researcher could introduce observer bias, although data collection and analysis were conducted independently to mitigate this risk. Future multi-center trials with larger and more diverse populations are needed to confirm these findings and assess long-term outcomes such as breastfeeding duration, maternal adaptation, and postpartum mental health.

Overall, this study provides robust evidence that integrating the MLCCM into maternity care can reduce childbirth fear, improve psychological outcomes, and encourage vaginal birth. Continuous and respectful midwifery care should be prioritized as a key component of maternal health policy, emphasizing prevention, empowerment, and emotional safety in childbirth.

## CONCLUSION

This randomized controlled study provides clear evidence that education and care provided within the MLCCM effectively reduce fear of childbirth, lower postpartum trauma symptoms, and increase vaginal birth rates among primiparous women. Continuous, woman-centered midwifery care fosters trust, emotional security, and empowerment, which together enhance women's confidence in their ability to give birth naturally.

These findings underscore the preventive and psychological benefits of continuity-based midwifery interventions. Integrating the MLCCM into national maternity services could help reduce unnecessary cesarean sections, improve maternal mental health, and promote respectful and individualized childbirth practices in Turkey.

Future research should explore broader applications of this model across different care settings and include long-term follow-up to assess its effects on maternal adaptation, bonding, and well-being. Strengthening midwife-led continuity of care represents a sustainable and evidence-based strategy for improving the quality and humanity of maternity services.

## ETHICS APPROVAL STATEMENT

This study received approval from the Non-Interventional Clinical Research Ethics Committee of Tarsus University (approval number: 128/64-2/12/2022). Institutional permission was also obtained from the Adana Provincial Directorate of Health and from Adana City Hospital, where the research was conducted.

## Data Availability

The datasets generated and/or analyzed during the current study are available from the corresponding author upon reasonable request.
